# Active dynamics of colloidal particles in time-varying laser speckle patterns

**DOI:** 10.1038/srep27681

**Published:** 2016-06-09

**Authors:** Silvio Bianchi, Riccardo Pruner, Gaszton Vizsnyiczai, Claudio Maggi, Roberto Di Leonardo

**Affiliations:** 1 Dipartimento di Fisica, Università di Roma “Sapienza”, Rome, I-00185, Italy; 2 CNR-NANOTEC, Soft and Living Matter Laboratory, Rome, I-00185 Roma, Italy

## Abstract

Colloidal particles immersed in a dynamic speckle pattern experience an optical force that fluctuates both in space and time. The resulting dynamics presents many interesting analogies with a broad class of non-equilibrium systems like: active colloids, self propelled microorganisms, transport in dynamical intracellular environments. Here we show that the use of a spatial light modulator allows to generate light fields that fluctuate with controllable space and time correlations and a prescribed average intensity profile. In particular we generate ring-shaped random patterns that can confine a colloidal particle over a quasi one-dimensional random energy landscape. We find a mean square displacement that is diffusive at both short and long times, while a superdiffusive or subdiffusive behavior is observed at intermediate times depending on the value of the speckles correlation time. We propose two alternative models for the mean square displacement in the two limiting cases of a short or long speckles correlation time. A simple interpolation formula is shown to account for the full phenomenology observed in the mean square displacement across the entire range from fast to slow fluctuating speckles.

The action of non-thermal fluctuating forces over small colloidal particles gives rise to non-equilibrium stochastic dynamics that display a wide and growing repertoire of peculiar behaviors. These active particles are usually self-propelled meaning that they are directly responsible for the force generation by exploiting an energy source that is distributed in the environment^1^. A large persistence time in these self-propelling forces is usually the key to the onset of non-equilibrium phenomena^2^ like rectification or phase separation in purely repulsive systems^3^,^4^. Most of these peculiar behaviors in active matter have the potential to enable new technologies like self-assembled micromotors^5^,^6^,^7^,^8^,^9^, targeted delivery^10^,^11^, and particle sorting^12^,^13^,^14^. In this context it would be highly desirable to find a general mechanism that could turn on activity in a wide range of otherwise passive colloidal objects. An attractive route is that of using dynamic light speckle fields as an active optical medium generating fluctuating forces with tunable intensity and correlations in space and time. Although Brownian motion in static random fields has been widely investigated theoretically^15^ and more recently with experiments in static speckle patterns^16^,^17^, much less is known for the dynamical case. In particular the fundamental problem of how the correlation time and length of the fluctuating force field determine the mean square displacement of a colloidal particle is still unclear. Douglass *et al*.^18^ used dynamic light scattering to study the mean square displacements of colloidal particles enclosed in a metallic cavity. An incident laser field is randomized by both reflections from the cavity surfaces and scattering by suspended colloids. The observed short time mean squared displacements evidenced a super diffusive behavior that was attributed to the action of optical forces fluctuating on a longer timescale. However the complexity of the system, where the particle themselves contribute to the fluctuations in the driving force field, and the limited number of control variable, basically the laser intensity alone, did not allow to investigate the full and richer range of possible transport regimes in optical active media. Using computer simulations Volpe *et al*.^19^ have found that the long-time diffusive behavior in a fluctuating speckle field depends on the interplay between the two characteristic time scales in the problem: the correlation time of the speckle intensity *τ*_*c*_ and the relaxation time *τ*_*r*_ of a colloidal particle in a local minimum of the potential associated to a static speckle configuration. As the ratio *τ*_*c*_/*τ*_*r*_ is increased, a transition from a superdiffusive to a subdiffusive behavior is observed with the diffusion coefficient reaching a maximum value at *τ*_*c*_/*τ*_*r*_ = 1. Interestingly, the same authors have shown that speckle patterns rigidly translated back and forth can be used for particle sorting in microfluidic device^20^.

In this work we use a spatial light modulator to generate one dimensional fluctuating random speckle patterns having a controllable correlation extent in time and space. We find that the mean square displacement of trapped colloidal particles always displays an exponential crossover from a short time regime that can be super or sub diffusive, to a purely diffusive regime at long times. The time scale of this crossover as well as the magnitude of the long time diffusion coefficient are controlled by the interplay between *τ*_*c*_ and *τ*_*r*_. Although an analytical solution of the full problem is probably unfeasible, we find approximate solutions in the two limiting cases of 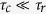 and 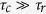. Our predictions for these two regimes agree quantitatively with experimental data while a simple interpolation between the two provides an excellent representation of the observed dynamical behavior throughout the entire *τ*_*c*_ range.

## Materials and Methods

Speckle patterns appear whenever multiple light waves interfere with random phases. This phenomenon can be easily observed when a coherent light beam is scattered by a rough surface or propagates through a disordered material. The statistical properties of the resulting random fields have been described widely in the literature^21^. Spatial light modulators (SLM) are dynamic diffractive elements that allow to apply a 2D array of arbitrary phase shifts to an incoming wavefront. When the SLM is placed in the back focal plane of a microscope objective, each pixel controls the phase of a different plane wave component on the object side. Speckle patterns can then be easily generated by applying random phase delays on the SLM. However, the intensity of the resulting speckle pattern will be spread over a large area and give rise to weak optical forces. By using a Gerchberg–Saxton algorithm^22^ it is possible to generate speckle patterns whose average intensity is concentrated over 1D or 2D subregions of the focal plane^17^. This allowed to study the effect of localization and transition to a long time diffusion regime in colloidal particles moving over static random energy landscapes.

### Speckle pattern generation

**Figure 1:**
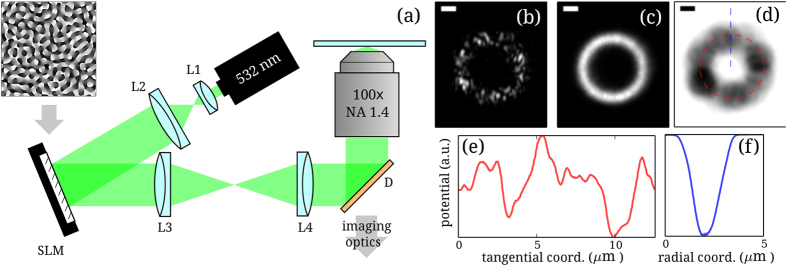
(**a**) Optical setup: the laser beam is expanded and modulated by an SLM placed in the Fourier plane of the objective. The phase modulation applied on the SLM produces on the objective focal plane a speckle pattern with desired profile. (**b**) Experimental realization of an individual ring-shaped speckle pattern obtained applying the phase mask shown in the top-left corner of (**a**). (**c**) Average intensity profile obtained by projecting sequentially 10^3^ patterns. (**d**) Potential seen by a 2 *μ*m bead estimated as described in the text. (**e**) Potential profile sampled along the entire circle shown as a red dashed line in (**d**). (**f**) Potential profile sampled along the radial coordinate (blue dashed line in (**d**)). Scale bars in (**b**–**d**) are 1 *μ*m.

**Figure 2:**
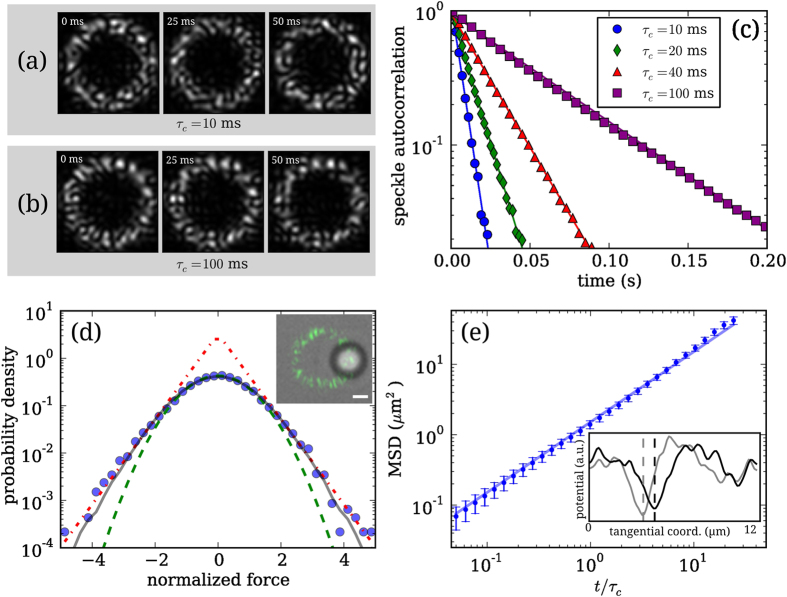
(**a**) A set of evolving patterns with small *τ*_*c*_. (**b**) A set of evolving patterns with large *τ*_*c*_ changes slower than in (**a**). (**c**) Measured time autocorrelation function of the intensity, lines are fits with exponential decay. The correlation time *τ*_*c*_ can be tuned as explained in the text. (**d**) Probability distribution of the force acting on a microbead subjected to a static speckle field (circles). Dashed line is a fit to a Gaussian while the dashed-dotted line is a fit to an exponential. Gray solid line plots the distribution obtained by taking the gradient of the potential which estimated as explained in the text. Inset depicts a 2 *μ*m silica bead trapped by the speckle pattern, scale bar is 1 *μ*m. (**e**) Mean square displacement, along the ring curvilinear coordinate, of the minima of the potential corresponding to an evolving speckle pattern. Error bars correspond to ±2 standard deviations. Inset shows a profile of an evolving potential at two distinct times (separated by *τ*_*c*_); the position of a minimum is highlighted by vertical dashed lines.

We use a custom optical tweezers setup^23^ (Fig. 1(a)). The laser beam (Coherent Verdi G2, *λ* = 532 nm) is expanded to illuminate a circle inscribed in the square active area of an SLM (Boulder Nonlinear Systems HSP256-0532, 256×256 pixels, switching time ≤2.8 ms). After being modulated the beam is coupled into an high numerical aperture objective (NA = 1.4) whose focal plane coincides with the Fourier plane of the SLM. In order to produce dynamical speckle fields, with controllable correlation times and a prescribed average intensity *Ι*(**r**) at the point **r** in the image plane, we generate a sequence of phase modulations given by:


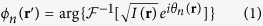


where *ϕ*_*n*_ is the *n*-th phase mask which is specified at each point **r**′ on the SLM, 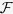 is the Fourier transform propagating the field from the SLM plane to the objective focal plane^24^. The phase term *θ*_*n*_(**r**) appearing in Eq. (1) is a matrix of independent random numbers uniformly distributed between 0 and 2*π*. Each array of phases *θ*_*n*_ produces a specific pattern 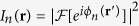 with same typical speckle size (which is of the order of *λ*/*NA* = 0.38 *μ*m). The average of the intensities *I*_*n*_(**r**) over many independent realizations of *θ*_*n*_ converges to the target intensity profile *I*(**r**). Figure 1(b) shows a single speckle pattern in the case of ring–shaped target intensity. The average obtained superimposing 10^3^ patterns converge to a smooth profile (Fig. 1(c)) with residual relative fluctuations of the order of 3%. When the random phases *θ*_*n*_(**r**) are updated with the rule *θ*_*n*+1_(**r**) = *θ*_*n*_(**r**) + *ϑ*(**r**), where *ϑ*(**r**) is a spatially uncorrelated random term with a Gaussian distribution of zero mean and variance 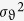, the sequence of speckle patterns will be exponentially correlated:


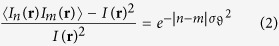


where the operator 〈·〉 denotes the average over many speckle pattern realizations. Projecting a sequence of phase modulations with a time delay Δ*t* will result in a correlation that decays as exp(−*t*/*τ*_*c*_) where 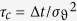. In our experiments we fix Δ*t* = 5 ms, corresponding to an SLM refresh rate of 200 Hz, and vary 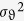 to tune the correlation time *τ*_*c*_. A sample of three consecutive speckle patterns is shown in Fig. 2(a,b) for a small *τ*_*c*_ (a) and for a large *τ*_*c*_ (b). We verified experimentally that the measured intensity time autocorrelation functions decay exponentially with the predicted values of *τ*_*c*_. Each curve in Fig. 2(c) was obtained computing the autocorrelation function at each pixel and then averaging over all pixels.

### Characterization of the force field

Finding the connection between the speckle pattern and the potential energy experienced by a colloidal particle is not an easy task in general. In the limiting case where the particle size is small compared to the wavelength (i.e. the Rayleigh regime) the conservative component of the force field is given by the gradient of the intensity which, in turn, is proportional to the potential energy^25^,^26^. When the particle size is comparable or larger than the wavelength, as in the present case, the calculation of the force field becomes computationally intensive^27^. However, a qualitative description of the expected potential landscape can be still obtained by assuming that each volume element of the bead is subject to the same potential that it would experience in the Rayleigh regime, i.e. proportional to the local light intensity. Within this approximation we completely neglect multiple-scattering and, by taking the continuum limit, the potential acting on the bead is approximated by convolving the speckle pattern with a disk having the same radius as the bead *a* = 1 *μ*m^16^,^28^. Figure 1(d) shows the estimated potential obtained by convolution of the pattern in (b). The resulting potential appears considerably smoother than the corresponding speckle intensity pattern and, in particular, no residual roughness is found along the radial coordinate that can be treated as a single potential well (Fig. 1(f)). The potential is however still rough along the azimuthal coordinate (Fig. 1(e)) displaying many minima separated by a typical distance *L* ≈ *a*. In the following we will therefore describe the system as having one degree of freedom, the curvilinear (angular) coordinate, that performs a stochastic dynamics over a fluctuating 1D potential.

Such an approximated description of the potential can be verified experimentally. To this aim we study one single 2 *μ*m diameter silica bead suspended in deionized water subjected to a *static* ring-shaped speckle field (see inset in Fig. 2(d)). When a static speckle field is projected onto the particle we observe that the bead is trapped in the nearest minimum. We project the speckle field onto an image plane that is far from the glass slide and the coverslip so that the hydrodynamic couplings with the walls are negligible (the distance from walls is always greater than 30 *μ*m). Moreover it has been demonstrated that, when considering only the in-plane motion of the colloid, the optical field is very well approximated by a conservative force **f**(**r**)^29^,^30^. In these conditions, by tracking the Brownian fluctuations of the microbead, we can extract the force by inverting the Boltzmann distribution:


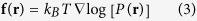


where *k*_*B*_ is the Boltzmann constant, *T* is the absolute temperature and *P*(**r**) is the probability distribution of the position of the particle.

To characterize the force field, we set the laser power to 70 mW (7 mW inside the sample) in such a way that the bead is barely trapped in a potential minimum; in this way the particle is able to explore as more as possible the potential around the minimum. We thus collect the trajectory of the particle in a static speckle pattern for a few minutes. We repeat this procedure for 10^3^ independent ring-shaped speckle patterns for an overall measurement time of several hours. On each trajectory we use Eq. (3) to extract the force field. The histogram of the force component tangent to the ring is shown in Fig. 2(d). Interestingly, the obtained distribution displays two different behaviors. A Gaussian shape is found at small *f* values while exponential tails appear at large force values. This non-trivial behavior can be closely reproduced by taking the gradient of the convoluted intensity pattern (black line) and rescaling it by a fitted scale factor. From the distribution of Fig. 2(d), and upon rescaling the power, we extract the variance of the force 〈*f*^2^〉 = 0.018 ± 0.001 pN^2^ corresponding to a laser power of 220 mW (22 mW inside the sample) that is kept fixed for all following experiments.

A crucial parameter in the following discussion is the typical relaxation time *τ*_*r*_ of a bead confined in a local minimum of the static potential. Calling *k* the typical stiffness of a local minimum, the relaxation time will be given by *τ*_*r*_ = (*μk*)^−1^. While the mobility *μ* = (6*πηa*)^−1^ depends trivially on the particle’s radius, the size dependence of *k* is hard to anticipate in an intermediate regime where the particle size is comparable to the wavelength^27^. Nonetheless, *τ*_*r*_ can be easily measured experimentally by tracking the motion of the bead in static speckle patterns. To this aim we project 10^3^ independent static patterns. For each pattern, we track the bead at a high-framerate (4 kHz) while fluctuating around a local potential minimum. From the obtained trajectories we compute the time autocorrelation function of position fluctuations *δx*(*t*) = *x*(*t*) − 〈*x*〉. Each autocorrelation function 〈*δx*(0)*δx*(*t*)〉 is fitted to the exponential 〈*δx*^2^〉*e*^−*t*/*τ*^, where both 〈*δx*^2^〉 and *τ* are fitting parameters. The average value of the fitted time constants is given by *τ*_*r*_ = 0.057 ± 0.002 s.

For reasons that will be clearer in the following section, we now characterize how minima in the potential energy landscape displace in time as the speckle pattern evolves. The potential is obtained, within our approximation, by convolving the speckle patterns as described at the beginning of this section. The convolution results in a smooth profile with no residual high frequency noise. In this situation the potential minima can be identified as those points where the first derivative of the interpolating function goes to zero and the second derivative is positive. We extract the positions of all minima in the 1D potential profile along the curvilinear coordinate (as the one shown in the inset of Fig. 2(e)). We then build the trajectories *y*(*t*) obtained by following the closest minimum in subsequent frames. Finally we compute the mean square displacements (MSD) as 〈Δ*y*(*t*)^2^〉 (where Δ*y*(*t*) = *y*(*t*) − *y*(0)) which displays a diffusive behavior as shown in Fig. 2(e). From a simple dimensional argument one can expect that *D*_*s*_ ≈ *L*^2^/*τ*_*c*_, given that the only relevant time and length scales are respectively *τ*_*c*_ and the the potential roughness *L*. Since *L* is approximately equal to the particle size *a* we have that *L*^2^ ≈ 1 *μ*m^2^ that is consistent with the measurement shown in Fig. 2(e) where we find *D*_*s*_ = 0.87 *μ*m^2^/*τ*_*c*_.

## Results

### Diffusion in dynamic speckle patterns

We turn now to the study of the colloidal dynamics in a time-varying speckle field. In this ring-shaped geometry, the micro-bead can diffuse indefinitely and thus we can average the dynamics for very long times. The bead is always illuminated, and therefore axially trapped, by multiple speckles (see Inset of Fig. 2(d)). Escapes from the ring are very rare (about one event every 3–4 hours) and usually occur in the beam propagation direction. While diffusing along the ring, the bead is strongly confined in the radial direction and only small fluctuations are observed (about 0.1 *μ*m). Since we are focused on the free diffusion in time-varying speckle fields we will ignore these fluctuations and consider only the 1D projection of the position of the particle along ring curvilinear coordinate.

**Figure 3:**
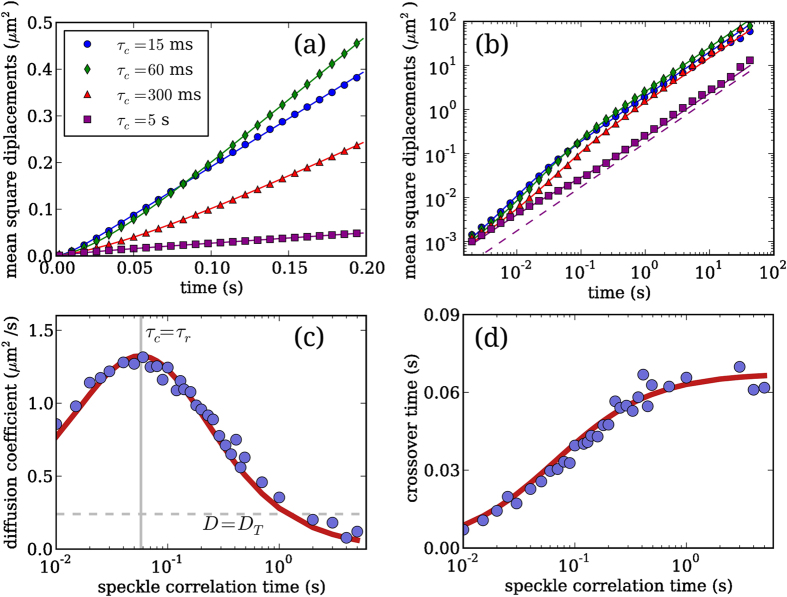
(**a**) Mean square displacements of a 2 *μ*m silica bead in dynamic speckle patterns with correlation times ranging from 15 ms to 5 s. Lines are fits with Eq. (5). (**b**) Same as (**a**) in a double log-scale. Dashed line plots the MSD of the potential minima corresponding to an evolving pattern with *τ*_*c*_ = 5 s. (**c**) Diffusion coefficient as function of the speckle correlation time (circles), the vertical line indicates the relaxation time of the bead in the speckle pattern. The thick solid red line represent a fit with Eq. (7). Horizontal dashed line indicates the thermal diffusion coefficient. (**d**) Crossover time between the non-diffusive regime and the diffusive one as a function of speckle correlation time (circles). Solid red line plots Eq. (8).

To cover different regimes we vary *τ*_*c*_ between 0.01 and 5 seconds that is, respectively, much shorter and much longer than the relaxation time *τ*_*r*_. For each value of the speckle correlation time *τ*_*c*_ we acquire 10 particle trajectories each lasting for 20 minutes for an overall time of about 200 minutes. The dynamic pattern decorrelates in a time *τ*_*c*_ meaning that for *τ*_*c*_ = 5 s, which is the largest value considered here, more than 10^3^ independent speckle patterns are displayed. In all experiments images are recorded at 500 fps. After image-processing and tracking we compute the mean squared displacement (MSD) of the colloid position along the curvilinear coordinate *x* of the ring i.e. 〈Δ*x*^2^(*t*)〉. For the sake of simplicity, we indicate with 〈·〉 both the average over time and over speckle realizations. Examples of the MSD obtained for small, intermediate and long *τ*_*c*_ are shown in Fig. 3(a,b).

Particle motions will be governed by an overdamped Langevin equation in the presence of a time-varying external optical force field *f*(*x*, *t*):


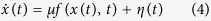


where *η*(*t*) is the thermal delta-correlated noise, i.e. 〈*η*(*t*)*η*(*t*′)〉 = 2*D*_*T*_*δ*(*t* − *t*′) with *D*_*T*_ = *μk*_*B*_*T*. An exact analytic approach to Eq. (4) is very hard, probably impossible, given the nonlinear and time dependent character of the external force. To make analytical progress we first analyse the two limiting cases 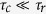 and 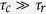. When 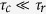 the external force decorrelates before the particle can explore the spatial structure of the field, which would take a time *τ*_*r*_. In other words, the force on the particle *f*(*x*(*t*), *t*) will decorrelate because of its explicit temporal dependence. We can then forget about the spatial dependence of *f* and describe it as force that fluctuate only in time with a correlation 〈*f*(*t*)*f*(*t*′)〉 = 〈*f*^2^〉 exp[−|*t* − *t*′|/*τ*_*c*_]. In this case the MSD can be obtained^31^:


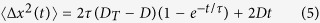


where *τ* = *τ*_*c*_ and *D* = *μ*^2^〈*f*^2^〉*τ*_*c*_ + *D*_*T*_. The MSD is diffusive at both short and long times with a diffusivity that is respectively *D*_*T*_ and *D*. A superdiffusive regime is present at the intermediate time scale *τ* being *D* > *D*_*T*_.

In the opposite regime 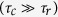 the particle has enough time to relax in the nearest minimum before the speckle fields decorrelates. Calling *y* and *k* respectively the position and the curvature of a local mimimum we assume that the optical force can be approximated by the linear form *f*(*x*) = −*k*(*x* − *y*). We further assume that the position of such a minimum diffuses with time. This last assumption is motivated by the result shown in Fig. 2(e) where the local mimima in the potential perform random displacements with variance 2*D*_*s*_Δ*t* where *D*_*s*_ ∝ 1/*τ*_*c*_. With the above assumptions Eq. (4) can be rewritten as a system of two coupled stochastic equations:


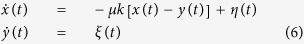


where 〈*ξ*(*t*)*ξ*(*t*′)〉 = 2*D*_*s*_*δ*(*t* − *t*′). Solving for the MSD (see^31^) we obtain the same form of Eq. (5) but with different parameters: *τ* = (*μk*)^−1^ = *τ*_*r*_ and *D* = *D*_*s*_. Again we find a purely diffusive regime at both short and long times but interestingly the intermediate time behavior goes from superdiffusive to subdiffusive as *D*_*s*_ becomes smaller than *D*_*T*_. A more refined model could also include fluctuations in *k* which might lead to small deviations from a purely exponential transient between the short and long time regimes. However, since the long time dynamics will be dominated by the diffusion of the minima we do not expect significant differences in *D*. Having found a MSD with the same form (5) in the two limiting cases 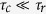 and 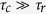 it is tempting to use the same expression as a fitting function throughout the entire *τ*_*c*_ range leaving *D* and *τ* as free fitting parameters. We find indeed that Eq. (5) fits very well the MSD data at small, intermediate and large values of *τ*_*c*_ as shown in Fig. 3(a,b). As expected from (5), the experimental MSD has an intermediate regime that goes from superdiffusive to subdiffusive upon increasing *τ*_*c*_. In Fig. 3(b) we also report, as a dashed line, the MSD of the potential minima as obtained in Fig. 2(e). As a further evidence supporting the proposed model, we find that the MSD of colloidal particles in slowly evolving speckles approaches the MSD of the minima at long times. The best fit values for *D* and *τ* are shown in Fig. 3(c,d). Note that when *D* ≈ *D*_*T*_ the non-diffusive term in Eq. 5 vanishes so that *τ* cannot be fitted, for this reason in Fig. 3(d) no data points are reported for 1 < *τ*_*c*_ < 3 s. Summarizing, the long time diffusivity *D* increases linearly with *τ*_*c*_ when 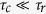 and decays as the inverse of *τ*_*c*_ for 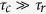. A simple formula that gives the correct limit expressions is provided by:


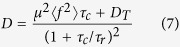


As shown in Fig. 3(c) this Eq. (7) fits very well the *D* data in the whole *τ*_*c*_ range explored. Moreover the fitting values of 〈*f*^2^〉 = 0.024 ± 0.002 pN^2^ and *τ*_*r*_ = 0.062 ± 0.006 s are in good agreement with the values measured independently in the static speckles as described above (〈*f*^2^〉 = 0.018 ± 0.001 pN^2^ and *τ*_*r*_ = 0.057 ± 0.002 s). Similarly, a straightforward interpolation can be found for *τ* which, in the two regimes 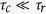 and 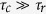 was found to be given respectively by *τ* = *τ*_*c*_ and *τ* = *τ*_*r*_:


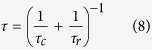


Figure 3(d) shows that Eq. (8) follows very well the data if *τ*_*r*_ takes the value previously obtained by fit shown in Fig. 3(c) (i.e. 0.062 ± 0.006 s).

## Discussion

We have studied the dynamics of colloidal particles driven by time-varying speckle fields with tunable correlation time. Starting from the limiting cases of fast and slow speckle correlation time we arrive to a general expression for the mean square displacement of colloidal particles as a function of the three parameters: speckle correlation time *τ*_*c*_, average relaxation time of a colloidal particle in a local energy minimum *τ*_*r*_, mean square optical force on a colloidal particle 〈*f*^2^〉. The obtained expression, described by Eqs (5), (7) and (8) well reproduces the observed mean square displacement in the entire range of *τ*_*c*_.

Besides describing the off-equilibrium dynamics of colloidal particles in time-varying speckle fields, our results could be relevant in understanding transport phenomena in active cellular environment^32^. Moreover, the possibility of driving interacting colloidal particles with tunable forces opens the way to a systematic experimental investigation of the statistical mechanics of active matter^3^,^33^.

## Additional Information

**How to cite this article**: Bianchi, S. *et al*. Active dynamics of colloidal particles in time-varying laser speckle patterns. *Sci. Rep.* **6**, 27681; doi: 10.1038/srep27681 (2016).
